# Systematic Review With Meta-Analysis: Diagnostic, Prognostic and Clinicopathological Significance of CircRNA Expression in Renal Cancer

**DOI:** 10.3389/fonc.2021.773236

**Published:** 2022-01-28

**Authors:** Wujun Wang, Shengfang Xie, Dongping Yuan, Dandan He, Liming Fang, Fengfeng Ge

**Affiliations:** ^1^ Jiangsu Province Hospital on Integration of Chinese and Western Medicine, Nanjing, China; ^2^ Nanjing University of Chinese Medicine, Nanjing, China

**Keywords:** circRNA, renal cancer, diagnostic, prognostic, clinicopathological, meta-analysis

## Abstract

**Background:**

Renal cancer (RC) is one of the most common malignant tumors of the urinary system, and molecular targets for the specific diagnosis and treatment of RC have been widely explored. The purpose of this study was to systematically analyze circular RNAs (circRNAs), which may serve as novel tumor markers in terms of the diagnosis, prognosis and clinicopathological characteristics of RC.

**Methods:**

PubMed and Web of Science were systematically searched for literature as up to July 30, 2021. All included studies were evaluated by the evaluation system, and the results were satisfactory. Hazard ratios (HRs) and odds ratios (ORs) were used to assess the association of circRNAs with diagnostic and clinicopathological indicators. The sensitivity (SEN), specificity (SPE), positive likelihood ratio, negative likelihood ratio, diagnostic odds ratio and area under the summary receiver operating characteristic curve (AUC) were combined to evaluate the diagnostic performance of circRNAs in RC.

**Results:**

We included 22 studies that met the criteria, including 18 that were prognostic, 4 that were diagnostic, and 12 that were clinicopathologically relevant. In terms of prognosis, we found that upregulated circRNAs were positively associated with poor overall survival in patients with RC (HR=1.63, 95% CI=1.43–1.85). In terms of diagnosis, the combined SEN, SPE and AUC of circRNAs in the diagnosis of RC were 0.82, 0.84 and 0.89 (0.86–0.91), respectively. In terms of clinicopathological features, upregulated circRNAs were associated with the Fuhrman grade (OR=0.641, 95% CI=0.471–0.873), T stage (OR=0.236, 95% CI=0.141–0.396), TNM stage (OR=0.225, 95% CI=0.158–0.321) and lymphatic metastasis (OR=0.329, 95% CI=0.193–0.560).

**Conclusion:**

Our meta-analysis confirms that circRNAs may be candidate biomarkers for the diagnosis, prognosis, and clinicopathological indicators of RC.

## 1 Introduction

Among tumors of the urinary system, renal cancer (RC) is characterized by a high incidence, high degree of malignancy, and poor prognosis (1, 2). According to the 2004 World Health Organization classification, RC can be divided into 10 types, including renal cell carcinoma (RCC), the most common type (3, 4). Distant metastases are found in approximately 1/3 of patients with RCC at the first visit, and the 5-year survival rate for patients with advanced RC is only approximately 20% (5). At present, the diagnosis of RC mainly depends on imaging, but it is difficult to distinguish it from a renal cyst or renal hamartoma in the early stage. In addition, the lack of specific serum markers makes early diagnosis more difficult (6). In terms of treatment, although targeted drugs, including sunitinib, have shown some success in treating RC, resistance remains a key issue to be addressed (7). Therefore, exploring the molecular basis and mechanism of the occurrence and development of RC, actively studying high-quality diagnostic markers of RC, and providing accurate therapeutic targets and prognostic indicators have become the primary tasks of current RC research.

Circular RNAs (circRNAs)—a particular type of endogenous noncoding RNA—were first identified in viruses in the 1990s (8). Initially, circRNAs were considered to be the product of missplicing. With the continuous progress of sequencing technology and molecular purification technology, an increasing number of studies have proven that circRNAs play different roles in the regulation of a variety of cell activities (9–12). Especially in recent years, a large number of experiments have proven that circRNAs play an important role in many fields, especially in tumor development. Many studies have demonstrated that different subtypes of circRNAs expressed in different tumors can play different inhibitory or promotive roles. In pancreatic cancer (PC), Yang et al. found that circRHOBTB3 was highly expressed in PC tissues and positively correlated with clinicopathological data, such as the clinical prognosis of patients. In terms of mechanism, circRNAs can promote the autophagy of PC cells and regulate the proliferation of PC cells by competitively binding miR-600 to upregulate NACC1. Similarly, a large number of circRNAs have been studied in RC (13, 14). For example, Cen et al. used bioinformatics to screen out the high expression of circSDHC in RC tissue. In subsequent clinical sample validation, it was further found that high circSDHC expression was closely associated with a poor patient prognosis and the TNM stage. Mechanistically, circSDHC can promote the proliferation and metastasis of RC cells *in vivo* and *in vitro* by downregulating miR-127-3p to promote the CDKN3/E2F1 signaling pathway (15). In addition, Frey et al. investigated the clinical value of 7 circRNAs in patients with RC. The results showed that circEGLN3, circEHD2, and circNETO2 may be used for the diagnosis of RC. Interestingly, high and low circEHD2 expression were also found to be independent predictors of the postoperative prognosis in patients with RC (16).

To determine whether circRNAs promote or inhibit RC, we summarized the results of different circRNA subtypes in RC based on current studies. Different software programs were used to meta-analyze the correlation of RC-related circRNAs with prognosis, diagnosis, and clinicopathology to explore the possible role and value of circRNAs in RC and to provide a reliable basis for the early diagnosis and precise treatment of RC in the future.

## 2 Materials and Methods

### 2.1 Literature Search Strategy

The literature search used in this paper was carried out in accordance with the preferred reporting items of the PRISMA statement standard. The article ran a search of related English language articles through PubMed and Web of Science until 30 July 2021 using the following related terms: (a) “renal carcinoma” or “kidney cancer” or “renal cancer” or “kidney neoplasm” or “renal neoplasm”; and (b) “circular RNA” or “circ RNA”. The literature search is performed independently by two researchers (WW and SX), who make appropriate assessments and data extraction. If there is any disagreement, discuss and decide with the third researcher (FG).

### 2.2 Inclusion Criteria and Exclusion Criteria

Studies concerning the value of circRNA expression in terms of the prognosis, diagnosis or clinicopathological characteristics of RC were eligible for quantitative synthesis. The inclusion criteria were as follows: (a) case-control study design; (b) diagnosis with RC by histopathology; and (c) confirmation of the relationship between circRNAs and the prognosis, diagnosis and clinicopathological characteristics of RC. The exclusion criteria were as follows: (a) ambiguous data or insufficient data leading to a study being defined as having unsuitable statistics; (b) reviews, comments, letters, or case reports; and (c) work unrelated to RC or circRNAs.

### 2.3 Data Extraction and Quality Assessment

The two researchers (WW and SX) separately extracted data from the included studies based on uniform criteria. Data extracted in terms of prognosis included the following: name of the first author; name of the circRNA; year of publication; country of origin; expression level in RC; cut-off value; sample size; detected sample; detection method; follow-up time; survival outcome; and survival analysis and hazard ratio (HR) with 95% confidence interval (CI) for overall survival (OS), progression-free survival (PFS), cancer-specific survival (CCS), metastasis-free survival (MFS) or disease-free survival (DFS). Data extracted in terms of diagnosis included the following: odds ratio (OR) and 95% CI used to summarize the above information and evaluate the accuracy of the diagnosis by the true positive (TP), false positive (FP), false negative (FN), true negative (TN) and area under the receiver operating characteristic (ROC) curve (AUC) in each study to synthesize the sensitivity (SEN), specificity (SPE), positive likelihood ratio (PLR), negative likelihood ratio (NLR) and diagnostic odds ratio (DOR). Data extracted in terms of clinicopathological characteristics included the following: gender; age; tumor size; Fuhrman grade; T stage; lymphatic metastasis; M stage; and TNM stage.

### 2.4 Statistical Analysis

The full-text data were statistically analyzed using Stata software (version 15.1). Each article was graded by Review Manager software (version 5.4), and only those meeting the criteria were included. In relation to the prognosis of patients, we mainly referred to the OS, PFS, CCS, MFS, and DFS curves in the study and extracted the relevant HRs and 95% CIs. The HRs and 95% CIs can usually be directly accessed from the study. When the prognosis was described as a Kaplan-Meier curve, we extracted the corresponding data using Engauge Digitizer version 4.1 and calculated the corresponding HRs and 95% CIs (17). In relation to the diagnosis of patients, the TP, TN, FP, and FN values were calculated from the known SEN and SPE values of the ROC curves using GetData Graph Digitizer software, and the NLR, PLR and DOR required for the results were further calculated (18). In relation to clinicopathological correlations, we used ORs and 95% CIs to analyze the clinical value of circRNAs associated with RC. An OR and 95% CI both > 1 suggested a positive correlation between the clinicopathological indicator and circRNA expression; otherwise, a negative correlation was indicated. In the heterogeneity test, I^2^ and Q tests were used to analyze the existence of heterogeneity. If I^2^>50% or P <0.1, heterogeneity was indicated. When the heterogeneity was obvious, a random-effects model was used; otherwise, a fixed-effects model was used (19). At the same time, we conducted a sensitivity analysis of all the included studies and excluded each individual study to compare its influence on the overall effect of the results of the meta-analysis. All tests were two-sided, and P < 0.05 was considered statistically significant. Publication bias was quantified by Egger’s test and Begg’s test and reflected by funnel plot analysis.

## 3 Results

### 3.1 Literature Retrieval and Screening

**Figure 1:**
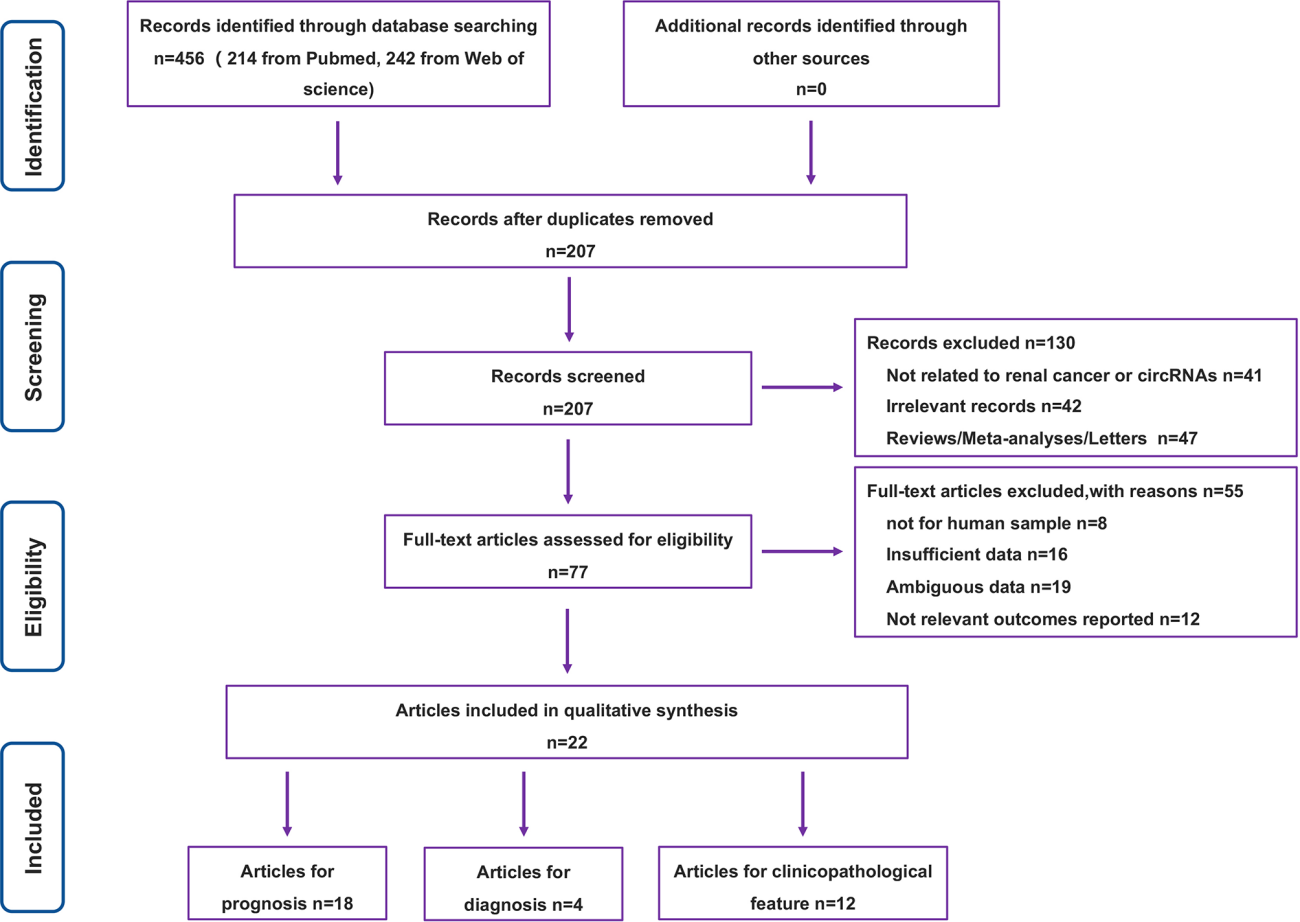
The PRISMA flowchart of the literature selection process.

The literature search involved in this paper and the process of filtering through relevant conditions is represented in **Figure 1**. A total of 456 studies were retrieved from the above database, and the remaining 207 studies were extracted after preliminary screening. Of the 207 studies, we also excluded studies unrelated to RC or circRNAs, irrelevant records, reviews and other studies that did not meet the requirements of format and content, leaving 77 studies. After the analysis and evaluation of the remaining 77 studies, we found that some studies did not meet the inclusion criteria, and a total of 22 studies were actually available for reference after elimination (15, 16, 20–39). Among them, 18 were related to prognosis, 4 were related to diagnosis, and 12 were related to clinicopathology.

### 3.2 Analysis and Evaluation of Research Results

**Figure 2:**
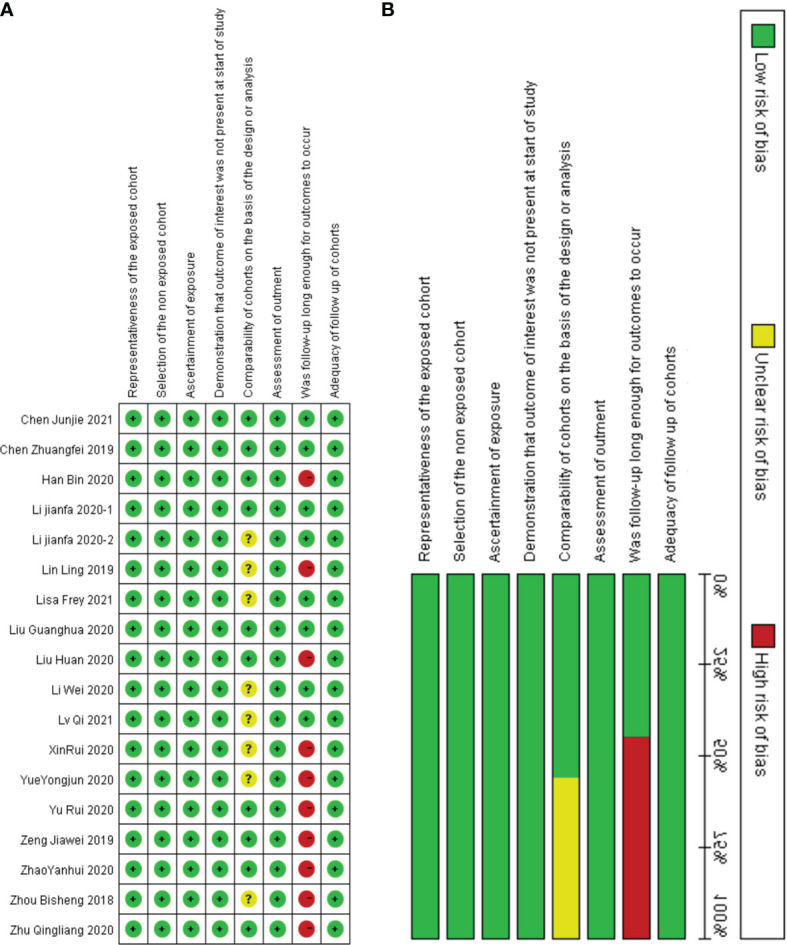
Quality assessment by the NOS. **(A)** each bias risk item for each included study; **(B)** each bias risk item is presented as a percentage for all included studies.

**Figure 3:**
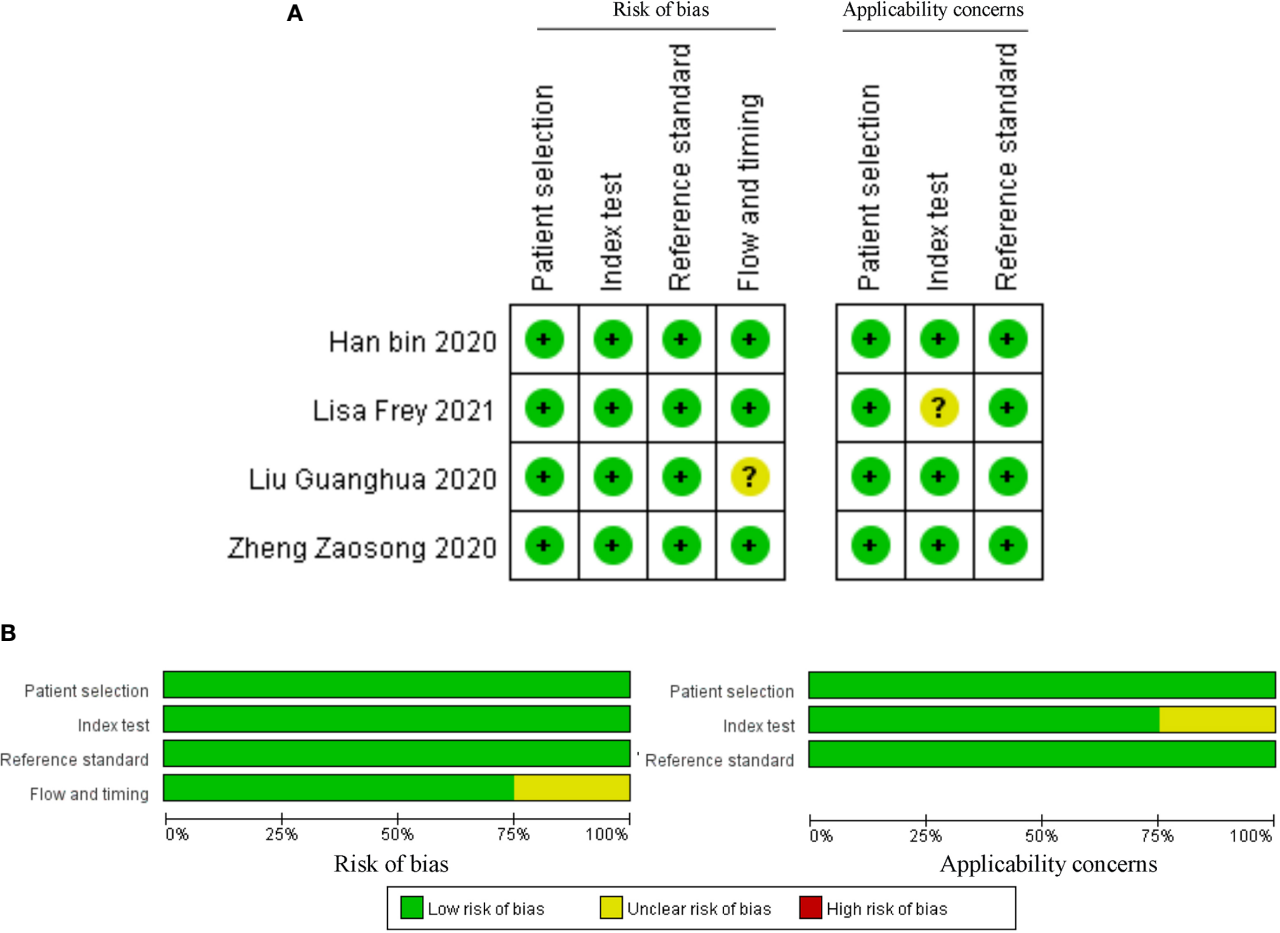
Quality assessment by the QUADAS II. **(A)** each bias risk item for each included study; **(B)** each bias risk item is presented as a percentage for all included studies.

The above studies were all published between 2019 and 2021, most of them were from China, and only one was from Germany. The minimum follow-up time was 40 months, and the longest was 240 months. A total of 22 circRNAs were mentioned, and the expression of circRNAs in RC was detected by quantitative real-time polymerase chain reaction (qRT-PCR). We found that the expression of all circRNAs in these articles was increased. The above research features are summarized in **Table 1**. We scored the prognostic and diagnostic components of the included studies by the Newcastle-Ottawa score (NOS) and the Quality Assessment for Studies of Diagnostic Accuracy II (QUADAS II) checklist, suggesting that the prognostic studies had scores ≥7 or the diagnostic studies had scores ≥4, which means that the methods and articles used in the studies were of high quality and could be used for reference (40, 41). The specific scores can be seen in **Figures 2** and **3**.

**Table 1:** Main characteristics of studies related to prognosis included in the meta-analysis.

No	Study	Circ	Year	Country	Cut-off	Sample size	Detected sample	Detection method	Follow up time	Survival outcome	Survival analysis	Variables				Reference
												HR^h^	95% CI^i^	p value	Obtained	
1	Cen Junjie	circSDHC	2021	China	Median	140	Tissue	qRT-PCR	99	OS**^a^**	Multiva**^f^**	2.916	1.508-5.637	<0.002	Direct	33468140
2	Liu Guanghua	circ0085576	2020	China	4.447	39	Tissue	qRT-PCR	80	OS	Multiva	1.372	1.077-5.151	<0.032	Direct	32541093
3	Lisa Frey	circEHD2	2021	Germany	Cutoff finder	101	Tissue	qRT-PCR	240	PFS**^b^**	Multiva	3.58	1.37–9.38	0.009	Direct	33946584
4	Lisa Frey	circEHD2	2021	Germany	Cutoff finder	101	Tissue	qRT-PCR	240	CCS**^c^**	Multiva	2.67	1.04–6.85	0.042	Direct	33946584
5	Lisa Frey	circEHD2	2021	Germany	Cutoff finder	101	Tissue	qRT-PCR	240	OS	Multiva	3.91	1.43–10.67	0.008	Direct	33946584
6	Lisa Frey	circNETO2	2021	Germany	Cutoff finder	101	Tissue	qRT-PCR	240	PFS	Multiva	0.17	0.06–0.50	0.001	Direct	33946584
7	Lisa Frey	circNETO2	2021	Germany	Cutoff finder	101	Tissue	qRT-PCR	240	CCS	Multiva	0.14	0.05–0.43	0.001	Direct	33946584
8	Lisa Frey	circNETO2	2021	Germany	Cutoff finder	101	Tissue	qRT-PCR	240	OS	Multiva	0.15	0.05–0.46	0.001	Direct	33946584
9	Zhou Bisheng	circPCNXL2	2018	China	Median	63	Tissue	qRT-PCR	60	OS	Univa**^g^**	1.87	1.07-3.28	0.028	KM**^j^**	30488762
10	Chen Zhuangfei	circ001895	2019	China	Median	60	Tissue	qRT-PCR	133	OS	Univa	1.23	0.77-3.72	0.0406	KM	31782868
11	Lin Ling	circEGLN3	2019	China	Median	80	Tissue	qRT-PCR	60	OS	Univa	1.04	0.65-2.13	0.035	KM	31904147
12	Li Wei	circPRRC2A	2020	China	NA	118	Tissue	qRT-PCR	100	MFS**^d^**	Multiva	1.53	1.47-3.814	0.032	Direct	32292503
13	Li Wei	circPRRC2A	2020	China	NA	118	Tissue	qRT-PCR	100	OS	Multiva	4.132	1.709-6.264	0.0009	Direct	32292503
14	Li jianfa	circMYLK	2020	China	NA	71	Tissue	qRT-PCR	80	OS	Univa	2.21	1.46-3.67	0.01	KM	32342645
15	Zhao Yanhui	ciRS-7	2020	China	NA	87	Tissue	qRT-PCR	100	PFS	Univa	1.03	0.45-1.78	<0.05	KM	32496306
16	Li jianfa	circTLK1	2020	China	NA	60	Tissue	qRT-PCR	100	OS	Univa	0.69	0.30-1.49	0.0074	KM	32503552
17	Li jianfa	circTLK1	2020	China	NA	60	Tissue	qRT-PCR	100	DFS**^e^**	Univa	1.29	0.63-5.13	0.0074	KM	32503552
18	Zeng Jiawei	circ001842	2019	China	Median	97	Tissue	qRT-PCR	60	OS	Univa	1.77	1.23-3.52	<0.05	KM	32729666
19	Yu Rui	circNUP98	2020	China	Median	78	Tissue	qRT-PCR	60	OS	Univa	0.89	0.66-2.13	<0.05	KM	32729669
20	Yu Rui	circNUP98	2020	China	Median	78	Tissue	qRT-PCR	60	DFS	Univa	1.32	1.01-3.22	<0.05	KM	32729669
21	Han Bin	circHIPK3	2020	China	Median	50	Tissue	qRT-PCR	60	OS	Univa	2.21	1.73-3.97	0.0191	KM	32821115
22	Zhu Qingliang	circAKT1	2020	China	Median	70	Tissue	qRT-PCR	60	OS	Univa	1.13	0.67-1.77	<0.05	KM	32900491
23	Liu Huan	circPTCH1	2020	China	Median	39	Tissue	qRT-PCR	40	OS	Univa	1.04	0.58-1.63	<0.05	KM	32929380
24	Xin Rui	circ001504	2020	China	NA	43	Tissue	qRT-PCR	60	OS	Univa	2.79	1.13-4.24	<0.05	KM	33110207
25	Yue Yongjun	circ101341	2020	China	Median	60	Tissue	qRT-PCR	60	OS	Univa	1.76	0.99-2.36	<0.05	KM	33408523
26	Lv Qi	circAGAP1	2021	China	Median	34	Tissue	qRT-PCR	150	OS	Univa	1.68	1.23-2.3	0.001	Direct	33618745

^a^OS, overall survival; ^b^PFS, progression-free survival; ^c^CCS, cancer-specific survival; ^d^MFS, metastasis-free survival; ^e^DFS, disease-free survival; ^f^Multiva, multivariate; ^g^Univa, univariate; ^h^HR, hazard ratio; ^i^95% CI, 95% confidence interval; ^j^KM, KM curve. NA, not available.

#### 3.2.1 Role of circRNAs in the Prognosis of RC

**Figure 4:**
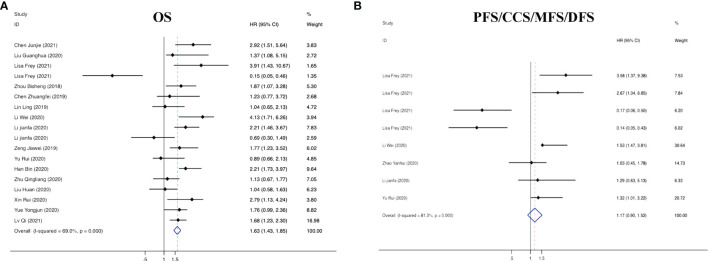
Forest plots of the **(A)** OS and **(B)** PFS/CCS/MFS/DFS of RC patients for circRNAs.

The characteristics of circRNAs related to the prognosis of RC are summarized in **Table 1**. In the 18 articles on prognosis we identified, we further found that all 18 circRNAs involved in the above studies were overexpressed in RC, and comprehensive evaluation of the OS was performed (HR=1.63, 95% CI =1.43–1.85, p<0.001, I^2 =^ 69.0%). These results suggest reduced OS in RC patients with elevated circRNA expression. In Lisa Frey’s study, although circNETO2 was highly expressed in RC tissues, it was negatively correlated with the risk of death (HR=0.15, 95% CI =0.05–0.46), suggesting that circNETO2 had an inhibitory effect on cancer. In addition, we summarized and analyzed the relationship between circRNAs and the PFS, CCS, MFS and DFS of patients with RC and extracted the corresponding data (HR=1.17, 95% CI=0.90–1.52, p<0.001, I^2 =^ 81.3%). The specific values are shown in **Figure 4**.

**Figure 5:**
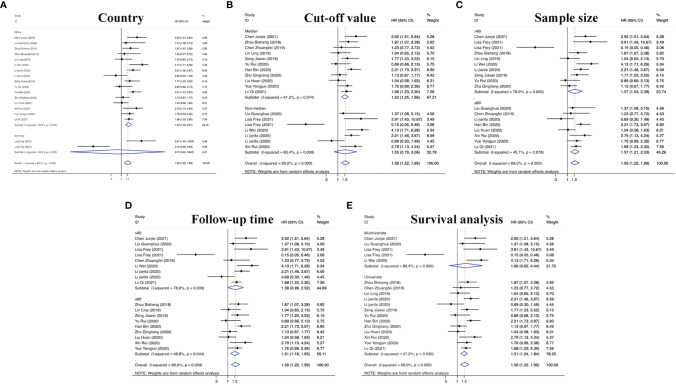
Subgroup analyses of OS for circRNAs, stratified by **(A)** country, **(B)** cut-off value, **(C)** sample size, **(D)** follow-up time, and **(E)** survival analysis.

**Figure 6:**
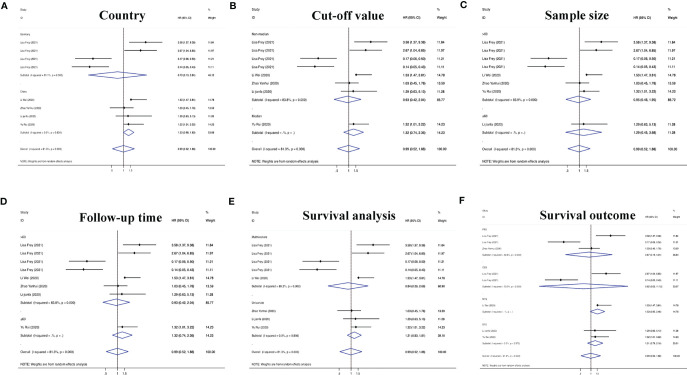
Subgroup analyses of PFS/CCS/MFS/DFS for circRNAs, stratified by **(A)** country, **(B)** cut-off value, **(C)** sample size, **(D)** follow-up time, **(E)** survival analysis, and **(F)** survival outcome.

On the basis of the above contents, we found that there was heterogeneity in the factors affecting the prognosis of patients with RC. Therefore, we conducted a subgroup analysis of the contents summarized in **Table 1** to further understand the specific factors affecting the OS of patients, as shown in **Figure 5**. We separately evaluated and analyzed factors such as the country of origin (China or Germany), cutoff value (median or nonmedian), sample size (>60 or ≤60), follow-up time (>60 months or ≤60 months), and survival analysis (multivariate or univariate). Similarly, we also performed a subgroup analysis of PFS, CCS, MFS and DFS to obtain the relevant data. The results are shown in **Figure 6**.

#### 3.2.2 Role of circRNAs in the Diagnosis of RC

**Figure 7:**
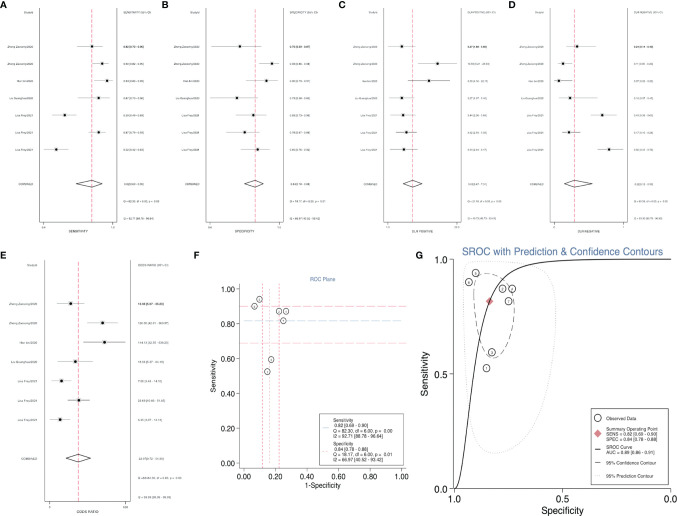
Forest plots of the combined **(A)** SEN, **(B)** SPE, **(C)** PLR, **(D)** NLR, **(E)** DOR, **(F)** ROC curve, and **(G)** SROC curve to illustrate the diagnosis of RC.

**Figure 8:**
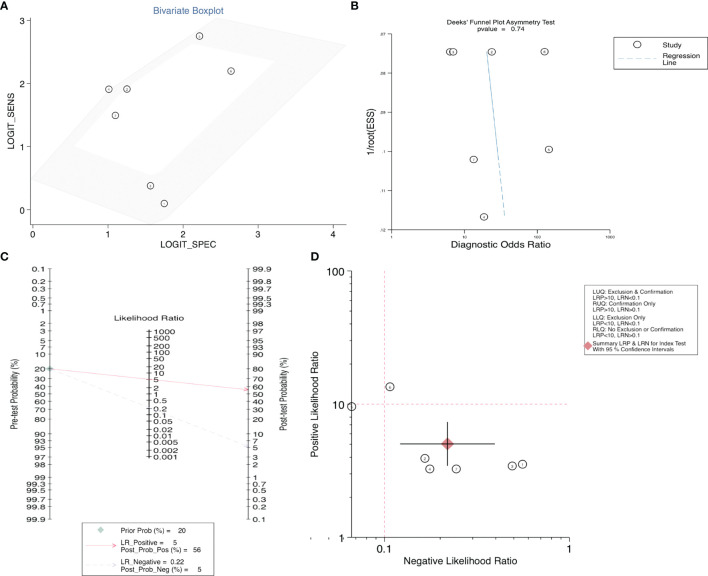
**(A)** Bivariate boxplot, **(B)** Deeks’ funnel plot, **(C)** Scatter plot, and **(D)** Fagan’s nomogram to illustrate the effect of circRNAs on the diagnosis of RC.

After accurate screening of the retrieved literature, we finally sorted out four articles related to diagnosis and scored the four articles using QUADAS II software, resulting in scores greater than 6 for all studies, which indicated high quality. The above articles were summarized, and corresponding data were extracted, including the SEN, SPE, PLR, NLR and DOR, as shown in **Table 2**. First, the SROC (summary ROC) curve was drawn, and the AUC was calculated to be 0.89 (0.86–0.91), as shown in **Figure 7G**. The results suggested that circRNAs had diagnostic significance in RC, and some known specific circRNAs could be detected for the diagnosis of RC. Moreover, we drew forest plots (**Figures 7A–E**) of the SEN (HR=0.82, 95% CI=0.69–0.90), SPE (HR=0.84, 95% CI=0.78–0.88), DLR (HR=5.03, 95% CI=3.47–7.31), NLR (HR=0.22, 95% CI=0.12–0.39) and DOR (HR=22.97, 95% CI=9.72–54.30). The ROC curve was drawn to analyze whether there was a threshold effect, and it was found that there was no shoulder arm shape, so it was considered that there was no threshold diagnostic effect (**Figure 7F**). To further analyze the clinical value of circRNAs associated with RC, Fagan’s nomograms and scatter plots of the PLR and NLR (**Figures 8C, D** **)** were plotted. When the probability was set to 20% before validation, the PLR of circRNAs as a diagnostic indicator increased to 56% after model correction, while the NLR decreased to 5%. In summary, circRNAs can play a very sensitive and accurate role in the clinical diagnosis of RC.

**Table 2:** Main characteristics of studies related to diagnosis included in the meta-analysis.

No	Study	Year	Country	Sample size		Detected sample	Variables										Reference
				Case	Control		AUC^a^	Sen^b^	Spe^c^	TP^d^	FP^e^	TN^f^	FN^g^	PLR^h^	NLR^i^	DOR^j^	
1	Lisa Frey	2021	Germany	101	81	Tissue	0.757	0.525	0.852	53	12	69	48	3.547	0.558	6.363	33946584
2	Lisa Frey	2021	Germany	101	81	Tissue	0.879	0.871	0.778	88	18	63	13	3.923	0.166	23.662	33946584
3	Lisa Frey	2021	Germany	101	81	Tissue	0.705	0.594	0.827	60	14	67	41	3.434	0.491	6.994	33946584
4	Liu Guanghua	2020	China	31	45	Tissue	0.844	0.868	0.730	27	12	33	4	3.215	0.181	17.779	32541093
5	Han bin	2020	China	50	50	Tissue	0.953	0.941	0.910	47	5	46	3	10.456	0.065	161.264	32821115
6	Zheng Zaosong	2020	China	90	90	Tissue	0.93	0.900	0.930	81	6	84	9	12.857	0.108	119.571	33453148
7	Zheng Zaosong	2020	China	60	40	Serum	0.86	0.815	0.740	49	10	30	11	3.135	0.250	12.538	33453148

^a^AUC, the area under the receiver operating characteristic curve; ^b^Sen, sensitivity; ^c^Spe, specificity; ^d^TP, true positive; ^e^FP, false positive; ^f^TN, true negative; ^g^FN, false negative; ^h^PLR, positive likelihood ratio; ^i^NLR, negative likelihood ratio; ^j^DOR, diagnostic odds ratio.

#### 3.2.3 Role of circRNAs in the Clinicopathologic Characteristics of RC

**Figure 9:**
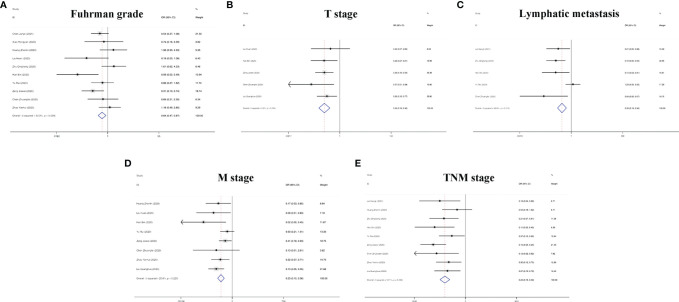
Forest plots of the clinicopathological characteristics, **(A)** Fuhrman grade, **(B)** T stage, **(C)** lymphatic metastasis, **(D)** M stage, and **(E)** TNM stage for circRNAs in RC.

**Figure 10:**
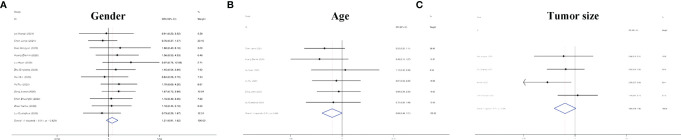
Forest plots of the clinicopathological characteristics, **(A)** gender, **(B)** age, and **(C)** tumor size for circRNAs in RC.

In addition to the role of circRNAs in evaluating the prognosis of RC patients and assisting in the diagnosis of RC, circRNAs are also closely related to many clinicopathological indicators of RC patients. In our meta-analysis, to quantify the relationship between circRNAs and the clinicopathologic characteristics of RC, we aggregated 12 studies with a total of 820 people (**Table 3**) and summarized the contents of the same influencing factor in different articles in the above 12 studies. The ORs (including 95% CIs) of different influencing factors were calculated and summarized. As shown in **Figure 9**, we found that high circRNA expression was correlated with a poor Fuhrman grade (OR=0.641, 95% CI=0.471–0.873), high T stage (OR=0.236, 95% CI=0.141–0.396) and TNM stage (OR=0.225, 95% CI=0.158–0.321), and the existence of lymphatic (OR=0.329, 95% CI=0.193–0.560) and distant metastasis (OR=0.235, 95% CI=0.154–0.359). In addition, we found no association of gender (OR=1.210, 95% CI=0.906–1.616), age (OR=0.693, 95% CI=0.476–1.011), or tumor size (OR=0.604, 95% CI=0.349–1.046) with high circRNA expression (**Figure 10**). Due to insufficient statistical data, some hematology-related influencing factors were not included in the table, such as the estimated glomerular filtration rate (eGFR), creatinine (Cr) level, and urine albumin creatine ratio (UACR), among others.

**Table 3:** Correlation between circRNAs and clinicopathological features of RC.

Upregulated circRNAs	No. of studies	No. of patients	Odds ratio (95%CI)	p value	Heterogeneity	I^2^ (%)
Gender (male/female)	12	820	1.210 (0.906-1.616)	0.829	6.61	0
Age (<60/≥60 years)	6	490	0.693 (0.476-1.011)	0.806	2.31	0
Tumor size (≤5/>5cm)	4	208	0.604 (0.349-1.046)	0.308	3.6	16.7
Fuhrman grade (I-II/III-IV)	10	709	0.641 (0.471-0.873)	0.034	18.09	50.3
T stage (T1-2/T3-4)	5	322	0.236 (0.141-0.396)	0.754	1.9	0
Lymphatic metastasis (-/+)	5	293	0.329 (0.193-0.560)	0.01	13.26	69.8
M stage (M0/M1)	8	547	0.235 (0.154-0.359)	0.223	9.44	25.8
TNM stage (I-II/III-IV)	9	613	0.225 (0.158-0.321)	0.334	9.1	12.1

### 3.3 Sensitivity Analysis

To evaluate the stability of our results, we performed a sensitivity analysis of the included studies by category, including prognosis (**Figures S1A, D**) and clinicopathologically related indicators (**Figure S2**). The results showed that excluding each study did not change the overall effect of circRNAs on the combination of HRs and ORs, suggesting that the conclusions of our meta-analysis were reliable.

### 3.4 Publication Bias

Egger’s and Begg’s tests were used to analyze the prognostic indicators of RC with high circRNA expression and evaluate whether there was obvious publication bias in the operating system. As shown in **Figure S1**, the results showed p > 0.05 for Egger’s and Begg’s tests of OS, as well as for the PFS, CCS, MFS, and DFS, indicating no significant publication bias. At the same time, Egger’s and Begg’s tests were carried out for the corresponding influencing factors, such as gender, age, tumor size, Fuhrman grade, T stage, lymphatic metastasis, M stage and TNM stage, and funnel plots were drawn. According to the obtained p values, there was no significant publication bias. Finally, in the diagnostic study of renal cancer-related circRNAs, bivariate and Deeks models (**Figures 8A, B** **)** were used to analyze the sensitivity, and no significant publication bias was found.

## 4 Discussion

As the third leading cause of death related to the urinary system, RC is difficult to detect in early stages, with a high metastasis rate and poor prognosis (42, 43). The advent of molecularly targeted drugs offers a novel approach for the treatment of patients with RC, with tumor-related signaling pathways being currently targeted, including VEGFR and mTOR inhibitors (44). Nevertheless, more than 30% of patients will likely become resistant to targeted drugs. Therefore, it is particularly important to comprehensively study the mechanism of the occurrence and development of RC as well as the molecules involved to generate precise treatment plans for different patients (45).

Many biomarkers have been found to be useful for the early diagnosis or long-term prognosis of patients with RC, such as mutation rates of VHL and PBRM1 in somatic cells, urine proteomics detection, and immune detection points (46–48). As a recent hot topic in tumor development, the role of non-coding RNA (ncRNA) in RC has been gradually explored (49). MiR-196a affects progression of RC by targeting BRAM1 to regulate the SMAD and MAPK signaling pathways, and miR-196 can be used as a prognostic marker for RC (50). Jasmine et al. also found 11 lncRNAs able to guide diagnosis of RC (51). As ncRNAs, circRNAs are formed *via* covalent bonding. With the rapid development of high-throughput sequencing and bioinformatics, the nature of circRNAs has been gradually revealed. Compared with mRNA, circRNA is more stable due to its closed ring structure, and it is not easily hydrolyzed by RNA enzymes. In addition, circRNAs formation is mostly driven by the exon lariat structure, and they are highly conserved and specific. Finally, circRNAs are widely present in exosomes and plasma, conferring distinct advantages as novel diagnostic markers (52–54).

According to defined inclusion and exclusion criteria, 22 studies of a total of 26 circRNAs associated with RC were analyzed. Regarding prognostic risk factors, 18 circRNAs were found to be associated with OS in RC patients, 3 circRNAs were associated with PFS, 2 circRNAs were associated with CCS, 2 circRNAs were associated with DFS, and 1 circRNA was associated with MFS (15, 16, 20–33, 36, 38). Overall, the risk of death in patients with high circRNA expression was 1.63 times higher than that in patients without high circRNA expression (95% CI =1.43–1.85). Interestingly, only Frey et al.’s study found that circNETO2 was highly expressed in RC, but prognostic analysis showed that circNETO2 may play a protective role in RC (16). No detailed experimental study has been conducted thus far, and determination of its mechanism may be a direction for the future development of targeted therapies. In the subsequent subgroup analysis, we further found that China as the country (HR=1.64, 95% CI=1.33–2.01), the median as the cutoff value (HR=1.52, 95% CI=1.25–1.86), a follow-up time ≤60 months (HR=1.51, 95% CI=1.18–1.93), univariate analysis (HR=1.51, 95% CI=1.24–1.84) and the sample size (HR=1.56, 95% CI=1.03–2.39) were risk factors affecting OS. However, there was no strong correlation between circRNAs and non-OS indicators in patients with RC (HR=1.17, 95% CI=0.90–1.52), which may be related to an insufficient sample size.

For RC diagnosis, although there were only 4 studies addressed this topic, 7 indicators were revealed, providing a valid basis for the diagnosis of RC patients (16, 27, 30, 37). Using SROC curve analysis, we calculated an AUC of 0.89 for circRNAs, indicating that 89% of our randomized RC patients had higher circRNA levels than the controls. The SEN was 82%, and the SPE was 84%. In addition, Fagan’s nomogram analysis of the clinical value of circRNAs can be used to indicate the sensitivity and specificity of a biomarker for the diagnosis of RC, and it showed positive predictive value. In addition, in our study, we listed some serum indicators, such as UACR, eGFR, Cr and cystatin C (CysC), to explore their relationship with the diagnosis of RC. However, due to the small number of studies involved and the lack of universality, they were not included in our study. In future studies, a composite evaluation index, rather than a single evaluation index, may be more valuable for the diagnosis of RC.

Finally, we performed a meta-analysis of clinicopathologic data from patients with RC, including a total of 840 participants in 12 studies (15, 21, 25, 27–32, 34, 35, 39). The results showed that high circRNA expression was consistent with a poor Fuhrman grade, lymph node metastasis, and poor clinicopathological features in patients with RC. In addition, other factors, such as age and gender. In conclusion, circRNA overexpression in patients with RC can indicate poor clinicopathological characteristics and can be used as a reference for clinical diagnosis and evaluation of therapeutic efficacy.

Of course, there are still many limitations to our research. On the one hand, although most studies have suggested that circRNAs have a cancer-promoting effect in RC, there are also a few contrary results, suggesting that there can be multiple effects of circRNAs. Second, the sample size of the diagnostic studies is relatively small, and most of them rely on the postoperative analysis of patient tissues. More circRNAs in preoperative samples of serum, urine and other body fluids from patients are expected to be studied. Finally, our study only examined overexpressed circRNAs, and the other side of the role of differentially expressed circRNAs in the occurrence and development of RC remains to be explored. Therefore, future efforts should be made to increase the quantity and quality of included studies to improve the accuracy and stability of the results.

## 5 Conclusion

In summary, our study found that the abnormal expression of circRNAs in patients with RC was closely related to its diagnosis, prognosis and clinicopathological features; that is, it was associated with the growth, progression and differentiation of RC tumors. Therefore, achieving an in-depth understanding of the biological and clinical effects of circRNAs, making efforts to screen potential targets for the treatment of RC, and developing new ideas for the diagnosis and treatment of RC will be the top priorities of future research.

## Data Availability Statement

The original contributions presented in the study are included in the article/**Supplementary Material**. Further inquiries can be directed to the corresponding authors.

## Author Contributions

WW and SX designed and carried out this study. WW and DY participated in collecting the literature on circ related to renal cancer. SX and DH participated in the analysis of clinicopathological features. WW and SX participated in the assessment of prognosis and diagnostic value. LF and FG were critically revised it critically for important intellectual content. All the authors approved the final version to be published.

## Funding

This study was supported by the Natural Science Foundation for Nanjing University of Chinese Medicine (XZR2020029), the Hospital Pharmaceutical Research Foundation for Nanjing Pharmaceutical Association and Changzhou Siyao Pharma (2019YX001), and the Second Batch of Science and Technology Projects for Traditional Chinese Medicine in Jiangsu Province (FY201805). The funder was not involved in the study design, collection, analysis, interpretation of data, the writing of this article or the decision to submit it for publication.

## Conflict of Interest

The authors declare that the research was conducted in the absence of any commercial or financial relationships that could be construed as a potential conflict of interest.

## Publisher’s Note

All claims expressed in this article are solely those of the authors and do not necessarily represent those of their affiliated organizations, or those of the publisher, the editors and the reviewers. Any product that may be evaluated in this article, or claim that may be made by its manufacturer, is not guaranteed or endorsed by the publisher.

## Supplementary Material

Supplementary Figure 1 Sensitivity analysis **(A, D)** and Publication bias judged by Egger’s **(B, E)** and Begg’s **(C, F)** funnel plots of circRNAs for the OS and PFS/CCS/MFS/DFS in RC.

Supplementary Figure 2 Sensitivity analysis of the circRNAs for **(A)** Fuhrman grade, **(B)** T stage, **(C)** lymphatic metastasis, **(D)** M stage, **(E)** TNM stage, **(F)** gender, **(G)** age, and **(H)** tumor size in RC.

Supplementary Figure 3 Publication bias judged by Egger’s funnel plots of the circRNAs for **(A)** Fuhrman grade, **(B)** T stage, **(C)** lymphatic metastasis, **(D)** M stage, **(E)** TNM stage, **(F)** gender, **(G)** age, and **(H)** tumor size in RC.

Supplementary Figure 4 Publication bias judged by Begg’s funnel plots of the circRNAs for **(A)** Fuhrman grade, **(B)** T stage, **(C)** lymphatic metastasis, **(D)** M stage, **(E)** TNM stage, **(F)** gender, **(G)** age, and **(H)** tumor size in RC.

## Abbreviations

RC, renal cancer; CircRNA, circular RNA; HR, hazard ratio; OR, odds ratio; SEN, sensitivity; SPE, specificity; PLR, positive likelihood ratio; NLR, negative likelihood ratio; DOR, diagnostic odds ratio; SROC, summary receiver operator characteristic; AUC, area under the SROC; OS, overall survival; RCC, renal cell carcinoma; PC, pancreatic cancer; CI, confidence interval; PFS, progression-free survival; CCS, cancer-specific survival; MFS: metastasis-free survival; DFS, disease-free survival; TP, true positive; FP, false positive; TN, true negative; FN: false negative; NOS, newcastle-ottawa score; QUADAS II, quality assessment for studies of diagnostic accuracy II; eGFR, estimated glomerular filtration rate; Cr, creatinine; UACR, urine albumin creatine ratio; CysC, cystatin C; ncRNA, non-coding RNA.
