# Supplementary figures and images for: Systematic Review With Meta-Analysis: Diagnostic, Prognostic and Clinicopathological Significance of CircRNA Expression in Renal Cancer
Source: Front Oncol. 2022 Jan 28;11:773236. doi: 10.3389/fonc.2021.773236 (PMC8832283; doi:10.3389/fonc.2021.773236)

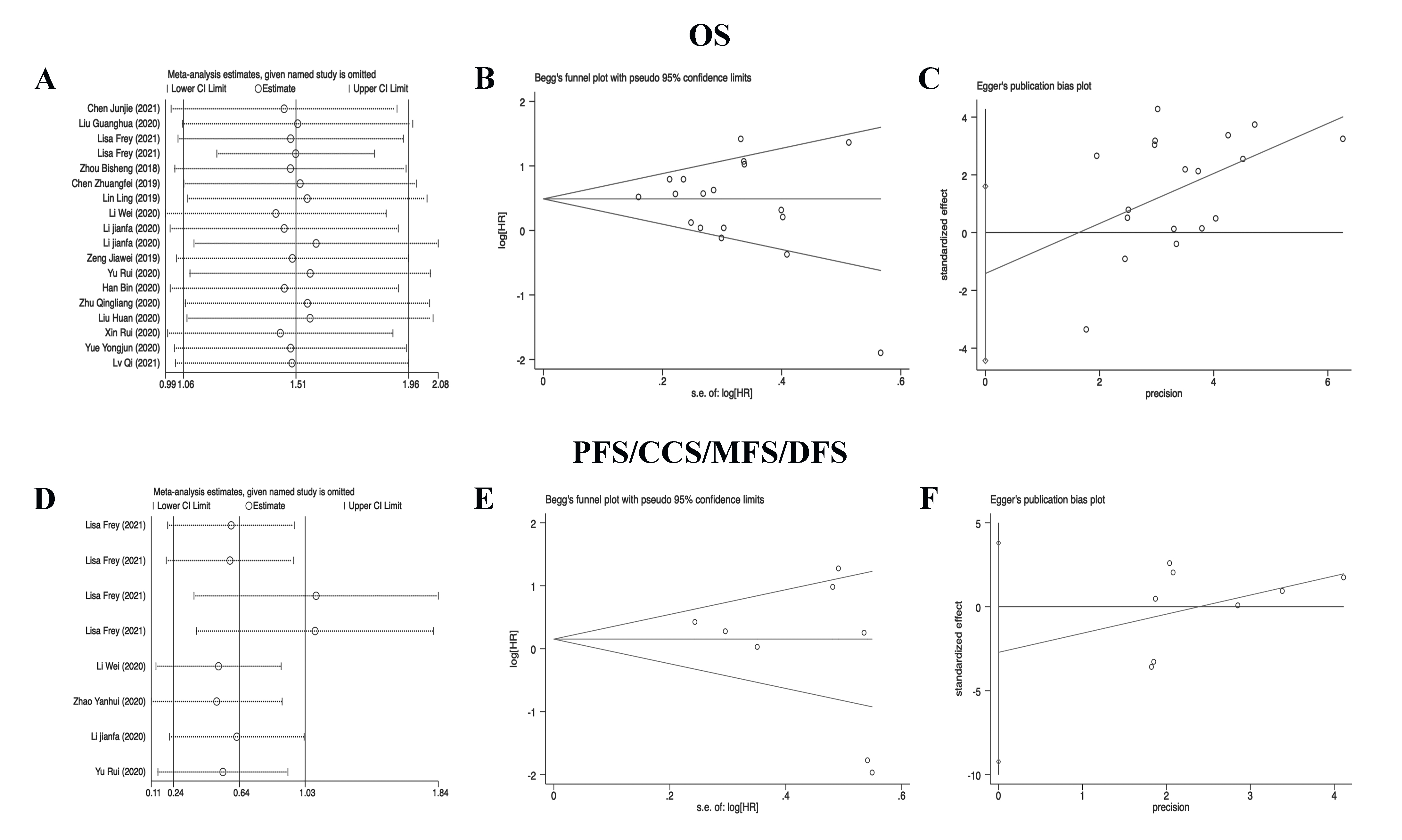

Supplement: Supplementary Figure 1 — Sensitivity analysis (A, D) and Publication bias judged by Egger’s (B, E) and Begg’s (C, F) funnel plots of circRNAs for the OS and PFS/CCS/MFS/DFS in RC. [file Image_1.jpeg]

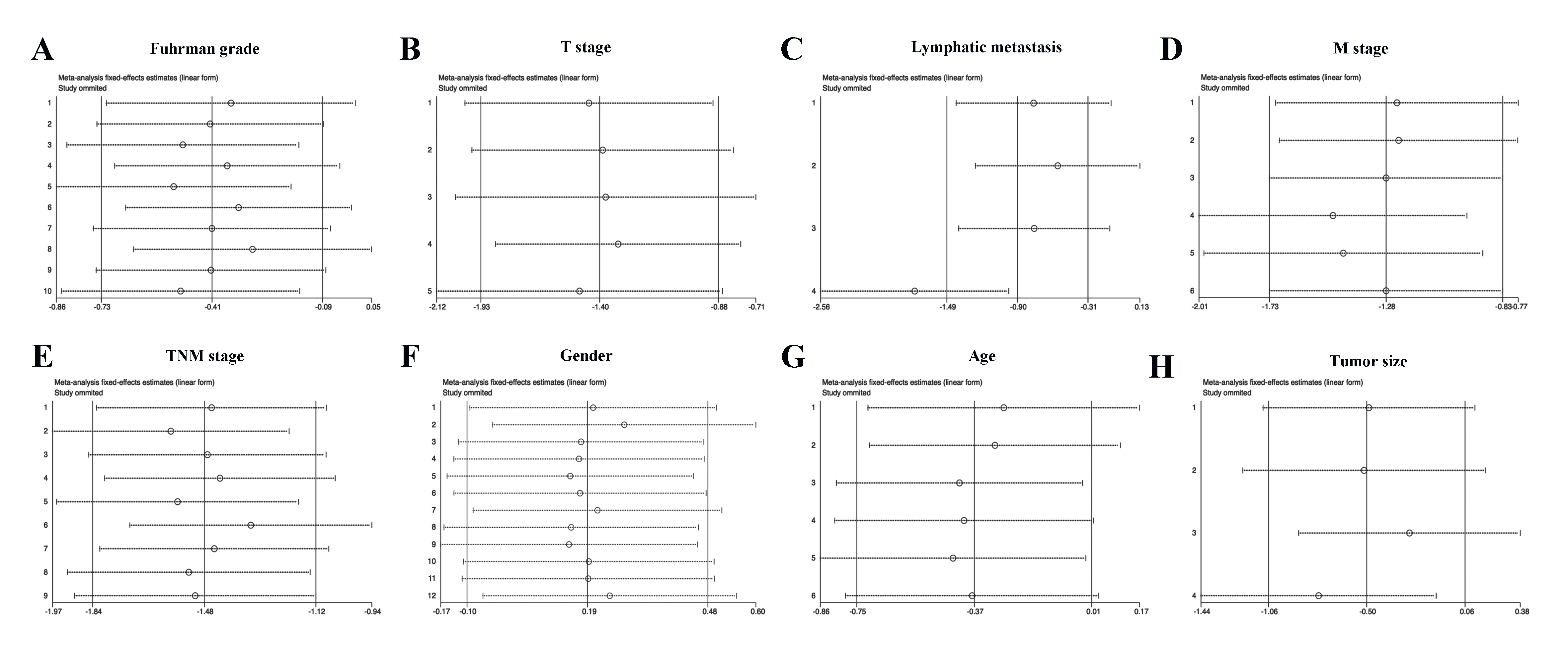

Supplement: Supplementary Figure 2 — Sensitivity analysis of the circRNAs for (A) Fuhrman grade, (B) T stage, (C) lymphatic metastasis, (D) M stage, (E) TNM stage, (F) gender, (G) age, and (H) tumor size in RC. [file Image_2.jpeg]

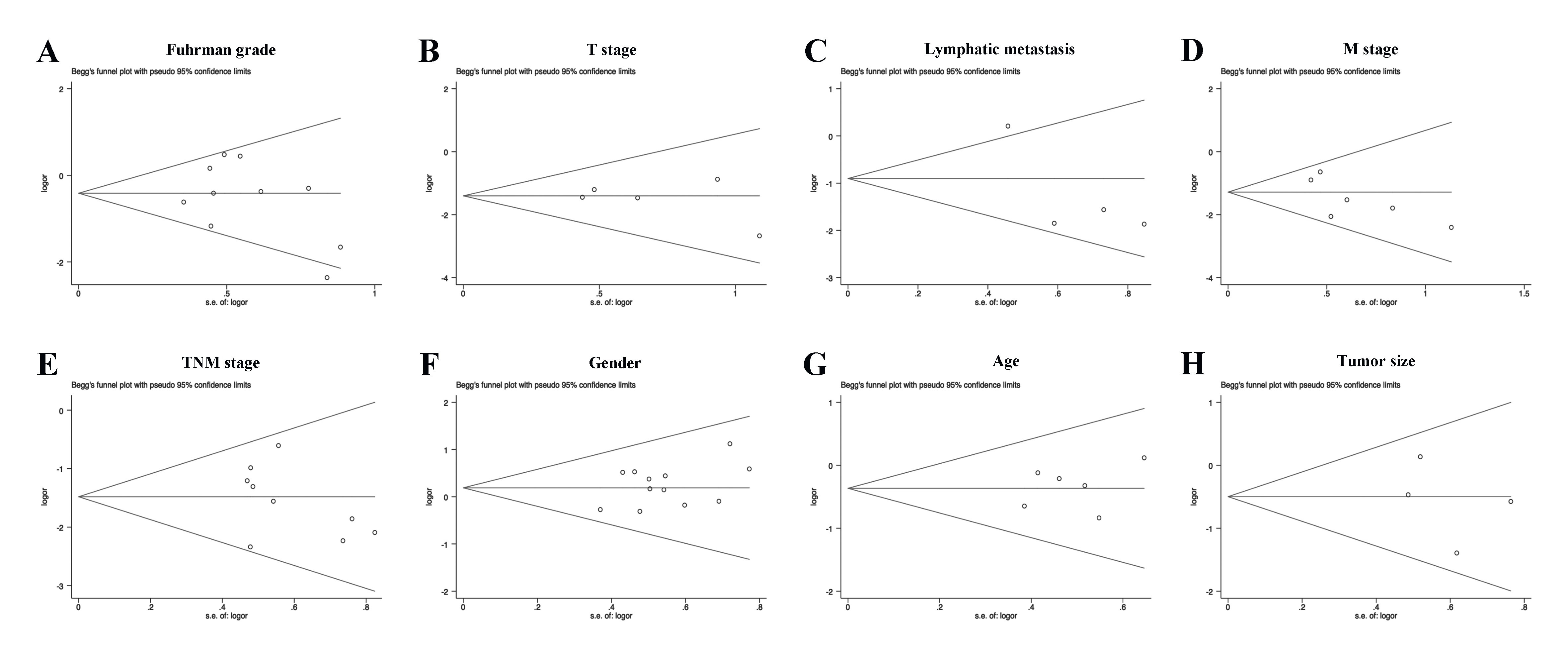

Supplement: Supplementary Figure 3 — Publication bias judged by Egger’s funnel plots of the circRNAs for (A) Fuhrman grade, (B) T stage, (C) lymphatic metastasis, (D) M stage, (E) TNM stage, (F) gender, (G) age, and (H) tumor size in RC. [file Image_3.jpeg]

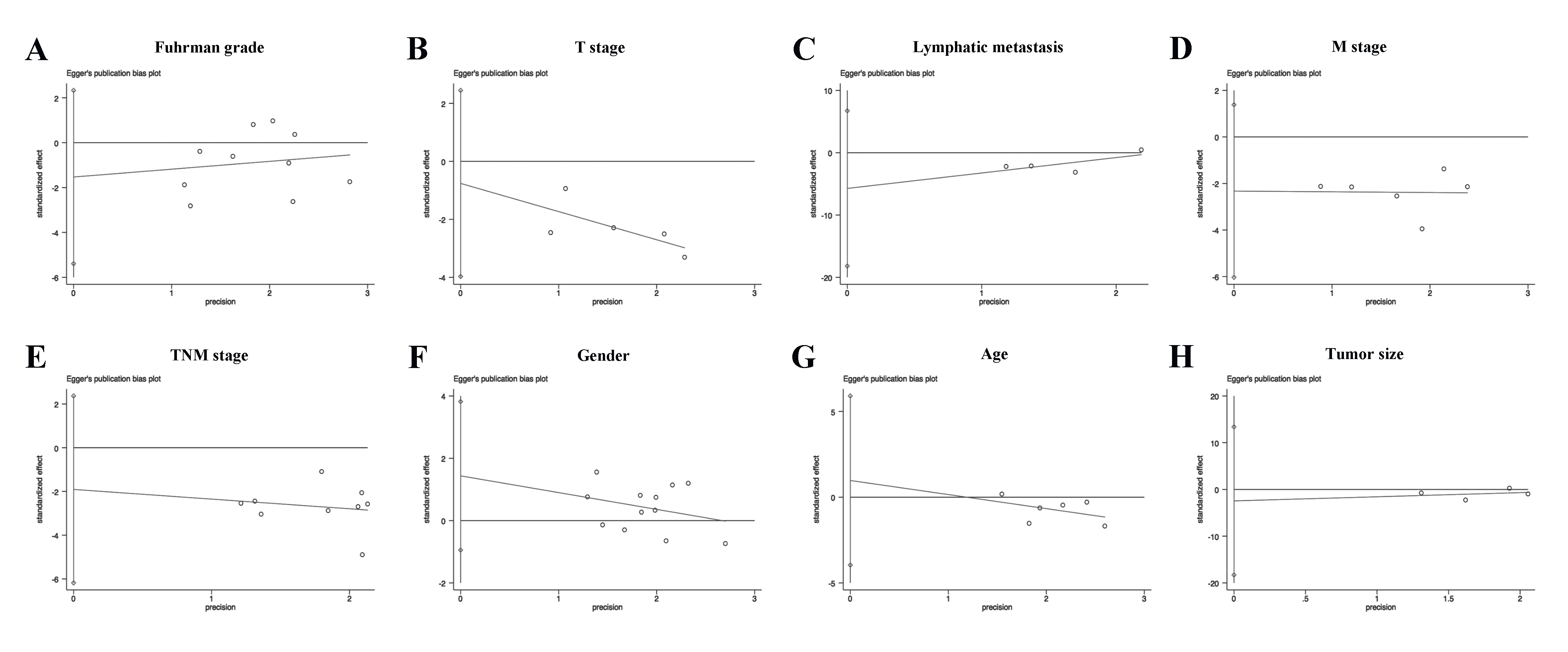

Supplement: Supplementary Figure 4 — Publication bias judged by Begg’s funnel plots of the circRNAs for (A) Fuhrman grade, (B) T stage, (C) lymphatic metastasis, (D) M stage, (E) TNM stage, (F) gender, (G) age, and (H) tumor size in RC. [file Image_4.jpeg]
